# Cardiac Rehabilitation and Complementary Physical Training in Elderly Patients after Acute Coronary Syndrome: A Pilot Study

**DOI:** 10.3390/medicina57060529

**Published:** 2021-05-25

**Authors:** Aurelija Beigienė, Daiva Petruševičienė, Vitalija Barasaitė, Raimondas Kubilius, Jūratė Macijauskienė

**Affiliations:** 1 Department of Rehabilitation, Medical Academy, Lithuanian University of Health Sciences, Eivenių g. 2, LT-50161 Kaunas, Lithuania; daiva.petruseviciene@lsmuni.lt (D.P.); vitalija.barasaite@gmail.com (V.B.); raimondas.kubilius@lsmuni.lt (R.K.); 2 Department of Geriatrics, Medical Academy, Lithuanian University of Health Sciences, Eivenių g. 2, LT-50161 Kaunas, Lithuania; jurate.macijauskiene@lsmuni.lt

**Keywords:** cardiac rehabilitation, exercise training, resistance training, balance training, ischemic heart disease

## Abstract

*Background and Objectives*: Nearly 23% of elderly patients hospitalized due to acute coronary syndrome have reduced muscle strength. It is assumed that these patients would better benefit from a complex training—a combination of endurance, strength, balance, coordination, and flexibility—in order to reduce the loss of muscle strength and mass and improve functional capacity. The aim of this study was to assess the effectiveness and safety of two different complementary resistance and balance training programs during short-term cardiac rehabilitation (CR) in elderly patients after a percutaneous or surgical intervention due to acute coronary syndrome. *Materials and Methods*: This randomized controlled trial was conducted from January 2020 to February 2021 in one Lithuanian rehabilitation hospital. A total of 63 participants who met the inclusion criteria were randomly assigned to three groups (at the ratio of 1:1:1): control (CG, *n* = 19), intervention 1 (IG-1, *n* = 26), and intervention 2 (IG-2, *n* = 18). All the patients attended a usual inpatient CR program of a mean duration of 18.7 ± 1.7 days, while the patients assigned to the intervention groups (IG-1 and IG-2) additionally received different resistance and balance training programs three days a week. Functional capacity, with 6-minute walk test (6MWT) and cardiopulmonary exercise testing (CPET), as well as physical performance, with the short physical performance battery (SPPB) test and one repetition maximum test (1RM) for leg press, were assessed at baseline and after CR. *Results*: The mean age of the participants was 72.9 ± 5.5 years; 73% were men. All parameters of functional capacity and physical performance improved significantly after CR (*p* < 0.05), except for peak VO_2_ that improved only in the IG-1. Comparison of CR effectiveness among the groups revealed no significant differences. *Conclusions*: All three rehabilitation programs were safe and well tolerated by elderly patients aged ≥65 years as well as improved functional capacity (6-minute walk distance and peak workload) and physical performance (SPPB and 1RM). Complementary resistance and balance training with traditional physical therapy means and exercises with mechanical devices did not show greater benefits for the results of physical performance compared with the usual CR program.

## 1. Introduction

Cardiac rehabilitation (CR) is a core element in the continuum of care for patients with cardiovascular diseases with the aim to stabilize and slow down disease progression, increase physical performance, reduce risk factors and mortality, and improve quality of life [1]. Therefore, participation in rehabilitation is considered an essential component of secondary prevention and is included in clinical guidelines for ischemic heart disease management across the world [1,2,3,4,5]. It is a complex, structured intervention of physical training, psychological and social support, and education, the cornerstone of which are aerobic exercises capable of improving physical performance but having only a negligible effect on muscle strength and mass of the extremities [6]. It is assumed that complex training—a combination of endurance, strength, balance, coordination, and flexibility exercises—might have greater benefits for elderly patients, striving to increase muscle mass and strength [7].

With aging, changes in body composition take place. After the age of 50 up to 70 years, people typically lose approximately 8% of muscle mass per decade. This has a huge impact on balance, gait, and overall capability to perform the activities of daily living [8]. These common, age-related changes in skeletal muscle mass are the main cause of impairment in physical function among elderly patients leading to greater risk of mobility disability, falls, and hospitalization [9,10].

One systematic review have demonstrated that multi-component training programs in combination with aerobic exercises with resistance and balance training, consisting of light-to-moderate workload exercises (from 30% of light to 60–70% of moderate maximal intensity) for at least eight weeks, have a positive effect on muscle strength and gait speed of elderly people [11]. Most commonly, traditional physical therapy tools, such as weights, elastic bands, and unstable platforms, are used in complementary exercises; however, scientific research that would use mechanical devices during CR to expect better results of physical performance is scarce. Therefore, the aim of this study was to assess the effectiveness and safety of two different complementary resistance and balance training programs during short-term CR in elderly patients after percutaneous or surgical intervention due to acute coronary syndrome.

## 2. Materials and Methods

### 2.1. Study Design and Participants

A clinical randomized controlled trial was conducted in a single rehabilitation center (Kulautuva Rehabilitation Hospital, Lithuanian University of Health Sciences, Kaunas, Lithuania) from January 2020 to February 2021. The study was approved by Kaunas Regional Biomedical Research Ethics Committee (permission No. BE-2-107, 19 December 2019) and was carried out following the guidelines of the World Medical Association Declaration of Helsinki. The trial was registered at ClinicalTrials.gov (No. NCT04768283).

All elderly patients referred to CR after percutaneous coronary intervention (PCI) or coronary artery bypass grafting (CABG) due to acute coronary syndrome were invited to take part in this study. The inclusion criteria were as follows: age ≥65 years; 6-minute walk distance (6MWD) ≥150 m; left ventricular (LV) ejection fraction ≥40%; and signed written informed consent. The exclusion criteria were as follows: complex CABG and valve surgery; severe cognitive impairment (mini-mental state examination score <18); implanted cardiac pacemaker; speech, vision, and hearing disorders; other severe concomitant diseases that would not allow active participation in the rehabilitation program and thorough examination.

### 2.2. Study Assessment

**Figure 1:**
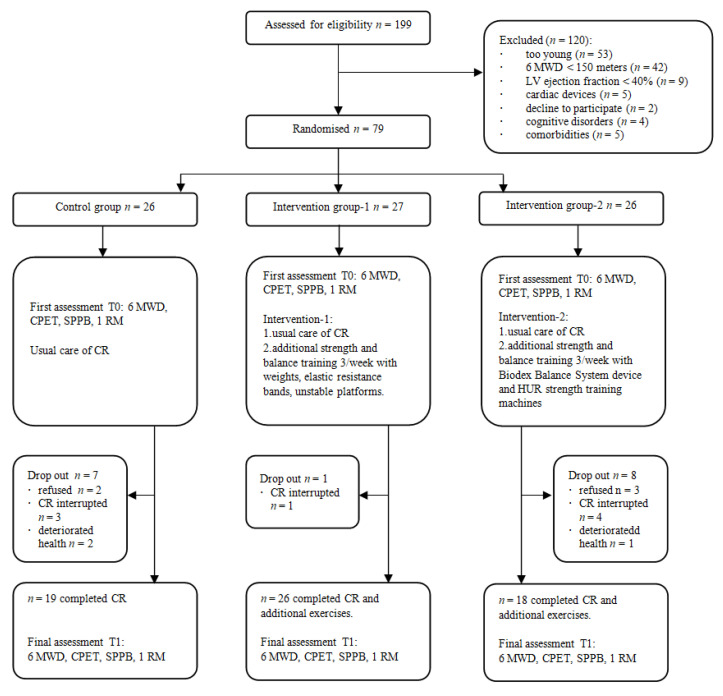
Study flow chart.

At the beginning of this study, a total of 199 elderly patients who arrived for CR were thoroughly assessed for eligibility to be enrolled in the study by a physical medicine and rehabilitation physician. Figure 1 depicts the flow chart of the study.

A total of 79 patients met the inclusion criteria. They signed the written informed consent, received a unique registration code, and underwent initial assessment. After initial assessment, participants were randomly assigned to three groups at the ratio of 1:1:1 using a computer-generated list: control (CG, *n* = 26), intervention 1 (IG-1, *n* = 27), and intervention 2 (IG-2, *n* = 26). During rehabilitation, eight participants dropped out due to rehabilitation discontinuation, four patients complained of deteriorated health and could not actively participate in training (deterioration was not related to the applied complementary physical training intervention), and five patients refused to undergo assessment (T1) after rehabilitation. After exclusion, the distribution of patients in the groups appeared to be as follows: 19, 26, and 18, in the CG, IG-1, and IG-2, respectively.

Participants were assessed two times: on admission to rehabilitation (T0) and after rehabilitation (T1). All assessments were conducted by the same team members as the rehabilitation: a physician and a physical therapist. During the study, anthropometric data, main disease and underlying conditions, and risk factors for cardiac diseases were evaluated. In addition, all the participants passed a comprehensive clinical evaluation of functional capacity and physical performance using the following tests: 6-minute walk test (6MWT) according to the recommendations by the American Thoracic Society [12]; the short physical performance battery (SPPB) [13]; evaluation of leg press one-repetition maximum (1RM) [14] by using strength training equipment (HUR, Finland); and cardiopulmonary exercise testing (CPET) (The CareFusion MasterScreen CPX, Germany) on a cycle ergometer [15] with the initial workload of 25 W. The workload of this testing latter increased by 12.5 W at each minute until the patient became exhausted—i.e., was unable to maintain the imposed pedaling speed of 60 revolutions per minute due to leg fatigue or dyspnea—or met the study discontinuation criteria. During CPET, peak workload (maximum watt) and peak oxygen consumption (VO_2_ mL/kg/min) were recorded.

### 2.3. Study Interventions

The participants underwent inpatient CR, the duration of which varied based on the diagnosis: 14 days for patients who suffered from unstable angina pectoris, 18 days for those who sustained acute myocardial infarction, and 20 days for patients after cardiac surgery. Moreover, they received the optimal medication treatment recommended by cardiologists, physical therapy, education on nutrition, management of risk factors, and psychological counseling. Before training, individual intensity and duration of aerobic training based on the initial assessment (T0) were tailored to each participant.

The conventional CR program includes breathing exercises (7 days a week, 15 min in duration) and aerobic training with an ergometer (Ergoline, Germany) (6 days a week, 30–50% watt maximum, 60–70% maximal heart rate (HRmax), up to 30 min in duration) [16].

The participants allocated to the intervention groups received, along with the conventional CR program, a complementary balance and resistance training three times a week:

(1) The IG-1 underwent a complementary training with traditional means of physical therapy. During warm-up, exercises improving coordination and maintaining the normal range of motion at moderate intensity were performed (10 repetitions, 3 sessions, score of 11 or 12 on the Borg’s scale). In the main part of the training, exercises improving the muscle strength of the upper and lower extremities by using elastic resistance bands (starting with a yellow resistance band, which provides the lowest level of resistance, and gradually increasing the resistance with red, green, and blue elastic bands) and weights (starting with a 0.5 kg weight and gradually increasing weights to 1 kg or 2 kg) at moderate intensity were performed (10 repetitions, 3 sessions, score of 12 to 15 on the Borg’s scale). Moreover, patients were involved in the dynamic balance training, at the beginning with their own body weight and later including unstable balance platforms (30 repetitions, 3 sessions, score of 11 or 12 on the Borg’s scale). During cool-down, stretching exercises were performed.

(2) The IG-2 underwent a complementary training with mechanical devices. The warm-up was the same as for the IG-1. In the main part of the training, balance tasks on the Biodex Balance System (USA) [17,18] in all 6 training modes for 3 min each at moderate intensity were accomplished (score of 11 or 12 on the Borg’s scale). In addition, exercises to improve the strength of the lower extremities on pneumatic technology-based HUR training devices at a moderate intensity of 30–50% of 1RM were performed (10 repetitions, 3 sessions with a 3 min break, score of 12 to 15 on the Borg scale). During cool-down, stretching exercises were performed.

Participants’ attendance in the physical therapist-led training was strictly registered; complementary exercises were performed in small groups up to three patients. During training, the heart rate of all participants was continuously monitored with a sensor (Polar, Finland). No adverse events associated with additional physical load were documented.

### 2.4. Statistical Analysis

Statistical analysis was performed with the Microsoft Excel 2013 and IBM SPSS Statistics 27.0 programs. Data were expressed based on their distribution: when the data were distributed non-normally, they were expressed as medians with interquartile ranges (IQR); when normally, then as means with standard deviations (SD). Categorical data were described by numbers and percentages. Three independent samples of normally distributed continuous data were compared using one-way analysis of variance (ANOVA), while the Kruskal–Wallis test was used to compare three independent samples of non-normally distributed continuous data. Categorical data were compared with the chi-square test; the Fisher exact test was used when the frequency in at least one cell was small. For comparison of two dependent samples of non-normally distributed continuous data, the non-parametric Wilcoxon signed-rank test was used. The level of significance was set at *p* < 0.05.

## 3. Results

Data of 63 patients with a mean age of 72.9 ± 5.5 years were analyzed. There were 73% of men. PCI was performed in 31 (49.2%) patients and CABG in 32 (50.8%). CABG was the most common surgery in the IG-1 compared with the IG-2 and CG (73.1% vs. 27.8% and 42.1%, respectively; *p* = 0.009). All the patients were diagnosed with hypertension and dyslipidemia and all who arrived in rehabilitation were given antiplatelet therapy with aspirin and clopidogrel or ticagrelor.

The groups were homogeneous by the sociodemographic and clinical data, except for age, CABG frequency, and CR duration: the patients in the CG were significantly older, the IG-1 had more patients who underwent CABG, and CR duration was longer by one day in this group; however, this had no impact on the results. There were no significant differences in other variables among the groups. The main characteristics of the study population are shown in Table 1.

### Effectiveness of Cardiac Rehabilitation

After CR, all study groups showed a significant improvement in all parameters of functional capacity and physical performance except for peak VO_2_, which significantly increased only in the IG-1. The main results of CR by the groups are summarized in Table 2. However, comparison of CR effectiveness among the groups showed no significant differences in any parameter of functional capacity and physical performance (Table 3).

## 4. Discussion

The aim of this study was to assess the safety of complementary resistance and balance exercise-based CR programs in elderly (≥65 years) patients who suffered from acute coronary syndrome and to evaluate their impact on physical performance and muscle strength. The main findings of this study showed that all three rehabilitation programs were safe and well tolerated by elderly patients as well as improved the parameters of functional capacity (6MWD, peak workload) and physical performance (SPPB, 1RM). We expected that exercises with traditional physical therapy tools would better improve patients’ functional capacity and physical performance than the usual CR program and that the training program with mechanical devices would have even a greater impact on the effectiveness of rehabilitation. Contrary to the expectation, participation in complementary exercises did not have any greater benefit regarding physical performance than the conventional CR program. These results could have been influenced by the small sample size—it was difficult to achieve an adequate sample size during the COVID-19 pandemic—or by the too short duration of the inpatient CR in Lithuania [19].

There is an increasing body of evidence that additional resistance exercises are safe and significantly improve functional capacity as well as increase muscle strength in patients with ischemic heart disease [20], chronic heart failure [21], and those who underwent CABG [22], and this is especially important for elderly patients.

Although a lot of studies are currently being conducted, authors use different physical exercise programs in an effort to find out the best one: of what intensity, frequency, and duration the CR should be and what type of exercises are most suitable for elderly patients. A recent study by Kim et al., published in 2020, reported that elderly patients needed CR of a longer duration than their younger counterparts: younger patients (<65 years) with acute myocardial infarction who had CR sessions 3 times a week showed an improvement in CPET parameters after 6 weeks, meanwhile in elderly patients (≥65 years), CPET parameters improved only after 12 weeks [23]. Other study showed a positive impact of a two month CR, given five times a week, on physical parameters such as 6MWD and VO_2_ in all age groups, even in very old (≥80 years) patients [24].

One of the first studies that confirmed the benefit of additional resistance and balance exercises, performed 5 days a week, for patients aged 75 years and more who underwent CABG, compared with usual CR program, was published in 2012 [25]. The additional exercises were similar to those in our study: balance training was performed by using unstable devices and balls, and for resistance training, weight machines and free weights were used (60% 1RM, 8–12 repetitions). The mean inpatient CR duration was 20 days, and the parameters of functional capacity—6MWD, Timed-Up-and-Go Test time, relative workload in W/kg—were significantly greater in the intervention group.

The main benefit of resistance training for elderly people is an improvement in muscle strength and endurance to prevent sarcopenia [26]. The results of our study are in agreement with the findings of the study published in 2016 [27]. This study demonstrated the effectiveness of inpatient four-week CR attended five days a week at an intensity corresponding to 60–70% of peak VO_2_ (score of 11 to 13 on the Borg’s scale) and additional exercises with weights and elastic bands, improving strength and flexibility, in elderly patients (>75 years). After CR, all indexes of physical performance (peak VO_2_, 6MWT, peak torque) improved considerably. In this study, 29% of the participants achieved more than a 15% increase in peak VO_2_ and such an increase was achieved by 28% of them in 6MWD and by 47.8% of them in muscle strength (peak torque). However, there was no control group; therefore, a comparison that could reveal if the additional exercises provided a greater benefit than CR was impossible.

There are some limitations of this study to be acknowledged: (1) the study was carried out in a single rehabilitation hospital; therefore, it is not clear if the results of this study could be generalizable to other populations; (2) the duration of CR was short and the mean duration of 18.7 ± 1.7 days might be insufficient to increase muscle strength at maximum, especially in elderly people; (3) a small sample size of this pilot study did not allow to reveal any significant differences when comparing the groups. Despite these shortcomings, the results of our study showed that elderly patients tolerated the complementary balance and resistance exercises well, and no adverse events related to training were documented. This demonstrates that resistance and balance training is safe for elderly patients after PCI or CABG.

## 5. Conclusions

Our study showed that all three rehabilitation programs were safe and well tolerated by elderly patients (≥65 years) with ischemic heart disease and that they improved functional capacity (6-MWD and peak workload) and physical performance (SPPB and 1RM). Complementary resistance and balance training—traditional means of physical therapy and exercises with mechanical devices—did not show a greater effect on the results of physical performance compared with the usual CR program.

## Figures and Tables

**Table 1:** Sociodemographic and clinical characteristics of the study population.

Characteristic	All (*n* = 63)	IG-1 (*n* = 26)	IG-2 (*n* = 18)	CG (*n* = 19)	*p* Value *
Age, years	72.9 ± 5.5	72.9 ± 4.4	70.1 ± 4	75.5 ± 6.9	**0.010**
Sex, *n* (%)					
Female	17 (27)	7 (26.9)	6 (33.3)	4 (21.1)	0.673
Male	46 (73)	19 (73.1)	12 (66.7)	15 (78.9)	
Height, m	1.7 ± 0.8	1.7 ± 0.7	1.7 ± 0.9	1.7 ± 0.8	0.268
Weight, kg	81.9 ± 14.2	82 ± 13.1	86.2 ± 17.5	77.6 ± 11.4	0.185
Body mass index, kg/m^2^	28.1 ± 3.7	28.7 ± 3.5	28.6 ± 3.8	26.9 ± 3.7	0.228
Current smokers, *n* (%)	5 (7.9)	2 (7.7)	2 (11.1)	1 (5.3)	0.858
LV ejection fraction, %	48.2 ± 5.1	49.6 ± 4.9	46.8 ± 4.2	47.6 ± 5.8	0.166
Initiation of CR after index event, days	9.7 ± 4	10.7 ± 4	9.2 ± 4.5	8.9 ± 3.2	0.256
CR duration, days	18.7 ± 1.7	19.5 ± 0.9	18.1 ± 1.8	18.2 ± 2.1	**0.009**
Comorbidities, *n* (%)					
Diabetes	11 (17.5)	7 (26.9)	1 (5.6)	3 (15.8)	0.184
Atrial fibrillation	12 (19)	5 (19.2)	3 (16.7)	4 (21.1)	0.944
Degenerative joint disease	5 (7.9)	1 (3.8)	3 (16.7)	1 (5.3)	0.265
Cancer	4 (6.3)	1 (3.8)	2 (11.1)	1 (5.3)	0.607
Treatment, *n* (%)					
Coronary artery by-pass graft	32 (50.8)	19 (73.1)	5 (27.8)	8 (42.1)	**0.009**
PTCA	31 (49.2)	7 (26.9)	13 (72.2)	11 (57.9)	
Medication, *n* (%)					
ACE inhibitor	49 (77.8)	17 (65.4)	15 (83.3)	17 (89.5)	0.126
Diuretic	42 (66.7)	20 (76.9)	11 (61.1)	11 (57.9)	0.343
Statin	62 (98.4)	25 (96.2)	18 (100)	19 (100)	0.485

Values are mean ± standard deviation unless indicated otherwise. * *p* value was determined by the chi-square test (for categorical data) or ANOVA (for normally distributed continuous data). Bold *p* values indicate statistically significant differences. IG-1, intervention group 1; IG-2, intervention group 2; CG, control group; LV, left ventricular; CR, cardiac rehabilitation; PTCA, percutaneous transluminal coronary angioplasty; ACE, angiotensin-converting enzyme.

**Table 2:** Effectiveness of cardiac rehabilitation in the groups, evaluating functional capacity and muscle strength.

Parameters	Intervention Group 1 (*n* = 26)		*p* *	Intervention Group 2 (*n* = 28)		*p* *	Control Group (*n* = 19)		*p* *
	T0	T1		T0	T1		T0	T1	
6MWD, m	285 (233; 316)	388 (338; 419.5)	**<0.001**	348.5 (336.5; 403)	434.5 (392.5; 475.2)	**<0.001**	319 (224; 406)	420 (350; 466)	**<0.001**
Peak workload, watt	64 (51; 74.7)	75 (59; 84.5)	**<0.001**	78.5 (58.2; 93)	93.5 (58.2; 107.7)	**<0.001**	85 (58; 98)	89 (57; 125)	**0.004**
Peak VO_2_, mL/kg/min	9.3 (8.4; 11.1)	11.2 (10.8; 12.3)	**0.009**	11.8 (8.8; 12.9)	12.3 (9.4; 15.3)	0.093	11.6 (9.3; 15.2)	12.1 (10; 14.3)	0.365
SPPB, score	9 (7.7; 10)	10.5 (9.2; 11.7)	**<0.001**	10.5 (9.7; 11.2)	11.5 (10; 12)	**0.006**	9 (8; 10)	11 (9; 12)	**<0.001**
Leg press 1RM, kg	31.5 (28; 48)	48 (41.2; 56)	**<0.001**	38 (31.7; 46.2)	55.5 (42.7; 56)	**0.001**	45 (33; 52)	50 (45; 65)	**0.001**

Values are medians (interquartile range). * *p* value by the Wilcoxon test. Bolded numerals indicate statistically significant differences. T0, baseline assessment; T1, assessment after CR; 6MWD, 6-minute walking distance; SPPB, short physical performance battery test; 1RM, one repetition maximum.

**Table 3:** Changes in functional capacity and physical performance during rehabilitation by the groups.

Parameters	Intervention Group 1 (*n* = 26)	Intervention Group 2 (*n* = 18)	Control Group (*n* = 19)	*p* Value *
	T1–T0	T1–T0	T1–T0	
6MWD, m	90.5 (46; 125.5)	66.5 (39; 76.7)	89 (29; 151)	0.317
Peak workload, watt	7.5 (4; 20)	13 (5; 21)	10 (4; 15)	0.596
Peak VO_2_, mL/kg/min	1.8 (0.4; 3.5)	1.75 (–0.5; 2.5)	0.55 (–0.47; 1.47)	0.103
SPPB, score	1 (0; 2)	1 (0; 1)	1 (1; 2)	0.295
Leg press 1RM, kg	14 (8.2; 16.7)	12.5 (3.7; 21)	8 (2; 15)	0.206

Values are medians (interquartile range). * *p* value by Kruskal–Wallis test. T1–T0, the change from baseline to the end of cardiac rehabilitation; 6MWD, 6-minute walking distance; SPPB, short physical performance battery test; 1RM, one repetition maximum.

## Data Availability

The data presented in this study are available on request from the corresponding author. The data are not publicly available due to ethical restrictions and data protection policies.
